# Projected Distributions of Two Key Vectors of Lumpy Skin Disease, *Aedes aegypti* and *Stomoxys calcitrans*, Under Climate Change

**DOI:** 10.1155/tbed/1457227

**Published:** 2025-09-09

**Authors:** Li Li, Zhulin Zhang, Haoyu Ran, Mingwei Xing, Boyang Liu

**Affiliations:** College of Wildlife and Protected Area, Northeast Forestry University, Harbin 150040, China

**Keywords:** biomod2, climate change, lumpy skin disease, species distribution model, vector

## Abstract

Lumpy skin disease (LSD), a severe transboundary disease of cattle, has caused substantial economic losses worldwide. Its transmission involves multiple vector species, among which *Aedes aegypti* and *Stomoxys calcitrans* are recognized as important contributors due to their broad distribution and ecological adaptability. Modeling the global distribution of the two key vectors is essential for anticipating their potential range expansion under climate change, thereby providing a scientific basis for developing targeted surveillance and control strategies for LSD. Our ensemble models revealed distinct environmental drivers and distributional responses for *A. aegypti* and *S. calcitrans*. The distribution of *A. aegypti* was predicted to be primarily influenced by urban land cover as well as temperature-related variables, especially the mean temperature of the wettest quarter (Bio8) and the mean temperature of the driest quarter (Bio9). In contrast, *S. calcitrans* was strongly driven by managed pasture coverage and precipitation seasonality, indicating its reliance on livestock-associated habitats and stable moisture conditions. Under future climate scenarios, *A. aegypti* showed a pronounced potential for expansion into higher latitudes, while *S. calcitrans* exhibited range shifts toward temperate regions. Taking the 2050s (SSP1-2.6) as an example, the percentage gain for *A. aegypti* reached 96.2%, while for *S. calcitrans*, the percentage gain reached 43.98%. Our findings highlight the importance of multiple vector assessments in predicting LSD risk under climate change. Distinct habitat shifts of *A. aegypti* and *S. calcitrans* indicate the need for differentiated control strategies in different regions.

## 1. Introduction

Lumpy skin disease (LSD), caused by the LSD virus (LSDV), is a highly contagious vector-borne disease that primarily affects cattle [1]. Since its first recognition in Zambia in 1929, [2] LSD has progressively spread beyond its original endemic areas in Africa to the Middle East, Europe, and, more recently, parts of Asia, including China and Southeast Asia [3, 4, 5]. The disease manifests as fever, nodular skin lesions, reduced milk production, and, in severe cases, death, resulting in substantial economic losses due to livestock productivity reduction, trade restrictions, and costs of disease control measures [6].

LSD transmission is closely associated with hematophagous arthropods, including biting flies and mosquitoes, which play a critical role in the mechanical or biological transmission of the disease [7]. Numerous studies have confirmed that *Aedes aegypti* and *Stomoxys calcitrans* serve as important vectors of LSD [8, 9]. The distribution and abundance of these vectors are strongly influenced by climatic factors such as temperature, humidity, and precipitation patterns. Particularly, humidity and ambient temperature play a decisive role in the activity and emergence of adult *S. calcitrans* [10, 11]. Climate change can alter these key environmental factors, potentially expanding their habitats into new regions [12]. Although vaccination is the primary strategy for controlling LSD outbreaks, its effectiveness can be limited by factors such as vaccine strain mismatch, incomplete coverage, and potential risk of attenuated vaccine [13]. For instance, vaccination with the attenuated Neethling strain has been reported to cause a slight skin lesion [14]. Vaccination alone cannot fully prevent virus transmission. Therefore, assessing the impact of climate change on vector dynamics is essential not only for understanding and predicting the future transmission risk but also for informing targeted vector management measures to complement vaccination efforts.

While species distribution model (SDM) has been widely used to predict the distribution of vector species under climate change scenarios [15–18], most studies have concentrated on a single vector and a single modeling algorithm [19–22]. Such approaches may overlook the complexity of multiple vector transmission, particularly the differential ecological niches that can influence disease dynamics. The absence of multiple vector assessments limits the comprehensiveness and applicability of risk prediction.

To address these limitations, we employed the biomod2 package (v4.2-6-2) to project the potential global distributions of *A. aegypti* and *S. calcitrans* under climate change. Biomod2 integrates multiple modeling algorithms (including maximum entropy [MaxEnt], generalized linear model [GLM], generalized additive model [GAM], generalized boosted regression model [GBM], RF, artificial neural network [ANN], flexible discriminant analysis [FDA], MARS, surface range envelope [SRE], XGBOOST, and classification tree analysis [CTA]), allowing for comparative evaluation of individual model performance and the generation of ensemble forecasts to improve prediction robustness, accuracy, and to reduce biases and uncertainties associated with single model approaches. This modeling framework enabled us to predict the potentially suitable habitats of the two vector species under different climatic scenarios, as well as their varying responses to environmental variables. Beyond their role in transmitting LSD, *A. aegypti* and *S. calcitrans* are also vectors of other human and animal pathogens (such as chikungunya, dengue, etc.) [23, 24].

## 2. Materials and Methods

### 2.1. Species Occurrence

The occurrence data for *A. aegypti* and *S. calcitrans* were obtained from three main sources: (1) Literature search: A systematic and comprehensive search was conducted using the keywords “*A. aegypti*/*S. calcitrans*” combined with “global distribution/occurrence/modeling distribution/investigation/capture/survey/monitor” in Google Scholar, PubMed, Web of Science, and China National Knowledge Infrastructure (CNKI). The search covered publications from 1970 to 2024. Only records with precise geographic coordinates were included. (2) Global Biodiversity Information Facility (GBIF): GBIF is an international network and data infrastructure providing a large number of species occurrence records worldwide. We searched and downloaded global occurrence data for *A. aegypti* and *S. calcitrans* from the GBIF database. (3) Walter Reed Biosystematics Unit (WRBU): The WRBU specializes in mosquito-borne diseases, and we downloaded the global occurrence data of *A. aegypti* from this source.

**Figure 1:**
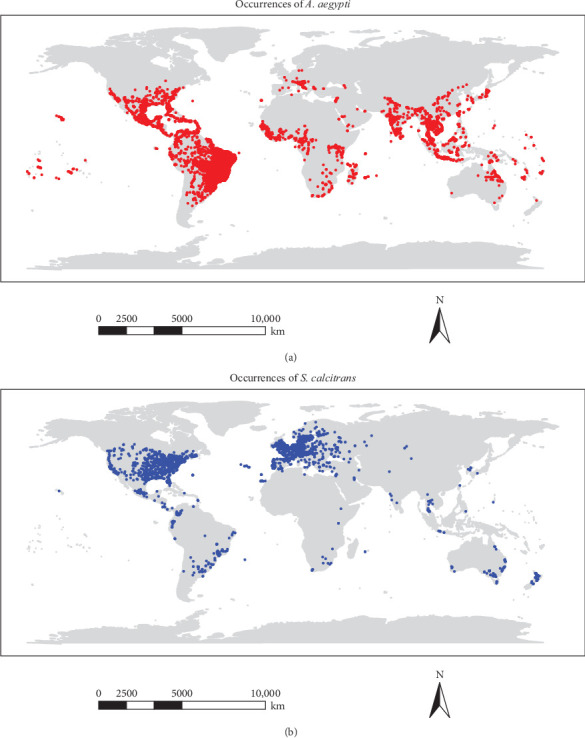
Geographical distribution of occurrence records for (a) *A. aegypti* and (b) *S. calcitrans*. The maps display the spatial locations of occurrence points used in the modeling process.

In total, 6777 occurrence records for *S. calcitrans* and 31,322 records for *A. aegypti* were collected from these sources. To address potential duplicates (i.e., records with identical coordinates reported in different sources or at different times) and to avoid over-representation of densely sampled locations, data cleaning was performed in R using the “dplyr” package (v1.1.4) and the “spThin” package (v0.2.0). Only the first record among duplicates was retained, and given our modeling resolution was set to 2.5 arc-min, we filtered the occurrence data to retain only a single record per grid cell. After filtering, 3181 occurrence points for *S. calcitrans* and 10,122 for *A. aegypti* were retained. The spatial distribution of these occurrence points was visualized using the “ggplot2” package (v3.5.1) and the “maps” package (v3.4.2) in R, and presented as occurrence maps for both species (Figure 1a,b).

### 2.2. Environmental Variables

Bioclimate variables, topographic, and land-use variables have been widely used in modeling the distribution of arthropod vectors [25–27]. In this study, a total of 19 bioclimatic variables, three topographic variables (elevation, slope, and aspect), and 14 land-use variables were considered for model construction.

The bioclimatic and land-use data were derived from the Coupled Model Intercomparison Project Phase 6 (CMIP6), which projects future climate patterns under different shared socioeconomic pathways (SSPs) representing various greenhouse gas emission trajectories and societal developments [28, 29]. We selected three SSP scenarios: SSP1-2.6, SSP2-4.5, and SSP5-8.5, representing low, intermediate, and high forcing pathways, respectively, with projected radiative forcing stabilizing at approximately 2.6, 4.5, and 8.5 W/m^2^ by 2100 [30]. Among the various general circulation models (GCMs) developed globally for climate change assessments, based on previous similar studies, we selected the BCC-CSM2-MR model developed by the Beijing Climate Center [31, 32]. Bioclimatic variables for both current and future scenarios at a 2.5 arc-min resolution were obtained from WorldClim (www.worldclim.org) [33]. The global land-use data, including 14 variables, were downloaded from Land-Use Harmonization (https://luh.umd.edu/data.shtml) dataset [34]. Slope and aspect data were extracted from elevation data provided by WorldClim.

In species distribution modeling, multicollinearity among environmental variables can significantly influence model performance [35, 36]. To address this, we first included all environmental variables in a preliminary modeling run to gain an initial assessment of variable importance. Then, we used the Pearson correlation method in the stats package (v4.3.3) in R to calculate pairwise correlations among all variables. Variables with a correlation coefficient greater than 0.7 were considered highly correlated and only one variable from each pair was retained according to ecological relevance and variable importance [37]. To further reduce multicollinearity, the variables retained from the correlation analysis were subjected to variance inflation factor analysis using the usdm package (v2.1-7) in R. Variables with VIF values over 10 were removed. The retained variables are shown in Table 1.

### 2.3. Modeling Procedure

SDMs were constructed using the biomod2 package (v4.2-6-2) in R (v4.3.3). Nine algorithms implemented in biomod2 were employed to construct the models: GLMs, GAMs, CTA, ANNs, SREs, FDA, random forest downsampling (RFd), GBMs, and MaxEnt. To enhance the robustness of the models, we followed the recommendation of the biomod2 team and generated pseudo-absence points using a random strategy, with 30,000 points for *A. aegypti* and 10,000 points for *S. calcitrans*. Pseudo-absence points were generated using a random strategy, with 30,000 points for *A. aegypti* and 10,000 for *S. calcitrans*. All models were built using a random cross-validation strategy, with 70% of the data in the training set and the remaining 30% in the test set. Model performance was evaluated using three metrics: the true skill statistic (TSS), the area under the curve (AUC) of the receiver operating characteristic (ROC), and Cohen's Kappa coefficient (KAPPA) [38, 39]. Since there is no universally accepted method for determining the optimal threshold, we established specific selection criteria for each species in this study [40–42]. For *A. aegypti*, only models that simultaneously achieved AUC ≥ 0.95, TSS ≥ 0.75, and KAPPA ≥ 0.75 were retained for ensemble modeling; for *S. calcitrans*, AUC ≥ 0.95, TSS ≥ 0.80, and KAPPA ≥ 0.78. During the modeling phase, the process was repeated 3 times, with models built independently based on the same dataset. The ensemble models were constructed using the weighted mean method, with prediction probabilities from the selected models weighted according to their TSS evaluation scores. Additionally, we assessed the importance of environmental variables in our models. It should be noted that, in this study, the projection phase involved high-resolution global raster data, which required substantial computational resources. To avoid memory overload, the ensemble models were constructed using only the best-performing models from a single modeling repetition.

### 2.4. Species Distribution Projection and Habitat Suitability Changes

Based on the constructed ensemble models, we predicted the current potential suitable habitats for *A. aegypti* and *S. calcitrans*. To assess potential changes in habitat suitability under climate change, we predicted the future potential suitable habitats under multiple climate scenarios, which combined three SSPs (SSP1-2.6, SSP2-4.5, and SSP5-8.5) across three future time periods (2050s, 2070s, and 2090s).

The continuous probability distribution maps generated by the ensemble models were converted into binary maps [43]. This binarization was performed using a threshold that maximizes the TSS. By comparing the current and future binary distribution maps, range change maps were generated for each future scenario relative to the current distribution. We further calculated the habitat areas of expansion, contraction, remain suitable, and remain unsuitable under each scenario.

## 3. Results

### 3.1. Model Performance Evaluation

Under the random cross-validation strategy, individual models were trained and evaluated for both species. Overall, the models demonstrated good performance, with evaluation metrics indicating some variability across algorithms. Based on evaluation scores, a subset of well-performing ones was selected for ensemble modeling. For *A. aegypti*, three algorithms were selected, including RFd, MaxEnt, and GBM, while four algorithms for *S. calcitrans* included RFd, FDA, GBM, and GLM.

The results showed that the ensemble models outperformed the individual models in terms of overall predictive accuracy and exhibited superior stability and generalization capability. Based on evaluation scores, the ensemble model for *A. aegypti* achieved a higher TSS (0.867) and KAPPA (0.868) than any individual model; similarly, the ensemble model for *S. calcitrans* also outperformed all single models with TSS (0.868) and KAPPA (0.868).

**Figure 2:**
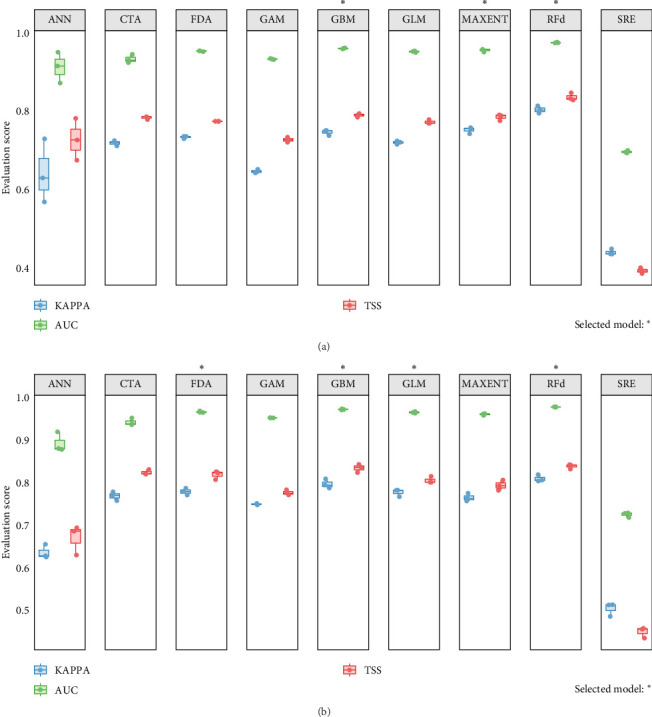
Performance evaluation of models for (a) *A. aegypti* and (b) *S. calcitrans* based on three evaluation metrics. Each boxplot summarizes the performance distribution derived from cross-validation runs for each algorithm (*⁣*^*∗*^ indicates algorithms selected in the ensemble model).

All cross-validation results were summarized and visualized (Figure 2a,b). The results showed the stability of model performance and the variability among algorithms.

### 3.2. Potential Suitable Areas of *A. aegypti* and *S. calcitrans*

**Figure 3:**
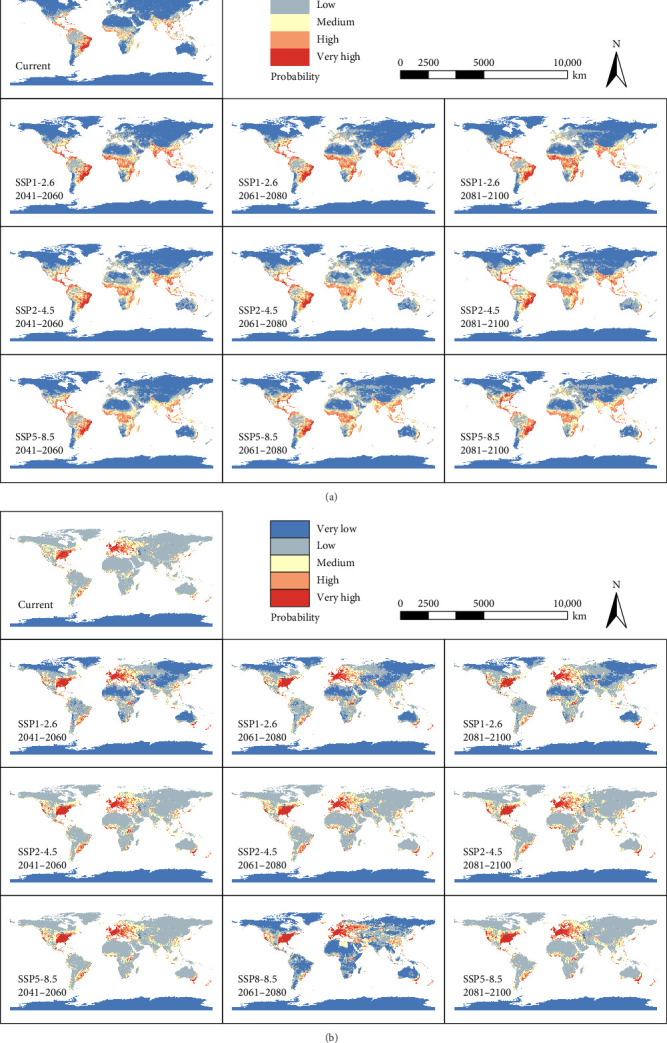
Predicted probability distributions of habitat suitability for (a) *A. aegypti* and (b) *S. calcitrans* under climate scenarios. Areas with higher suitability are shown in red, while those with lower suitability are shown in blue.

We visualized the potential global distribution of *A. aegypti* and *S. calcitrans*, and the suitability maps demonstrated the extent and intensity of habitat suitability for both species (Figure 3a,b), highlighting geographic regions where environmental conditions are most favorable for their persistence. In the maps, areas of high suitability are shown in red, while low suitability areas are indicated in blue.

Under current climatic conditions, the predicted high suitability areas for *A. aegypti* are primarily concentrated in tropical and subtropical regions. These regions include large parts of South America, especially Brazil and its surrounding countries, and Central America. Western, central, and parts of eastern Africa show high suitability. In Asia, suitable areas cover Southeast Asia, including India, Thailand, Vietnam, Malaysia, southern China, and parts of the Philippines and Indonesia. Additionally, some areas of eastern Australia and the southern parts of the United States. These areas generally have warm temperatures, high humidity, and a favorable precipitation pattern that supports the survival and reproduction of *A. aegypti*.

For *S. calcitrans*, the results exhibit high suitability areas primarily in temperate and subtropical regions of the Northern Hemisphere. Suitable areas are concentrated in central and eastern North America, particularly the southeastern United States. In Europe, there is a large area of high suitability spanning from western to eastern Europe. Parts of North Africa and the Middle East also show moderate suitability. In Asia, suitable areas are scattered across southeastern China, Japan, the Korean Peninsula, and parts of India and Southeast Asia. In the Southern Hemisphere, suitable areas include parts of Brazil, Argentina, southern Africa, southeastern Australia, and New Zealand. Suitable habitats are mostly located in regions with temperate climates, high livestock density, and sufficient humidity that provides suitable conditions for growth and reproduction.

### 3.3. Suitable Habitat Shifts Under Climate Change Scenarios

Projections under different future climate scenarios indicate significant changes in suitable habitat distribution across the globe.

Based on the continuous probability distribution maps under future climate scenarios, *A. aegypti* is projected to exhibit a poleward shift in the distribution of suitable habitats, with an expansion into higher latitude regions. Meanwhile, some currently highly suitable areas show a slight contraction in habitat suitability, particularly in regions such as India, southern China, and parts of Southeast Asia, where climate changes make the conditions less favorable for *A. aegypti*.

For *S. calcitrans*, future projections indicate a general shift and expansion of suitable habitats toward higher latitudes under all climate change scenarios. The extent of highly suitable areas is predicted to increase in temperate regions, particularly in northern Europe and parts of North America. These regions become more suitable due to climate change. Meanwhile, some current suitable regions in lower latitudes may experience a reduction in suitability, particularly in areas projected to become hotter and drier.

**Figure 4:**
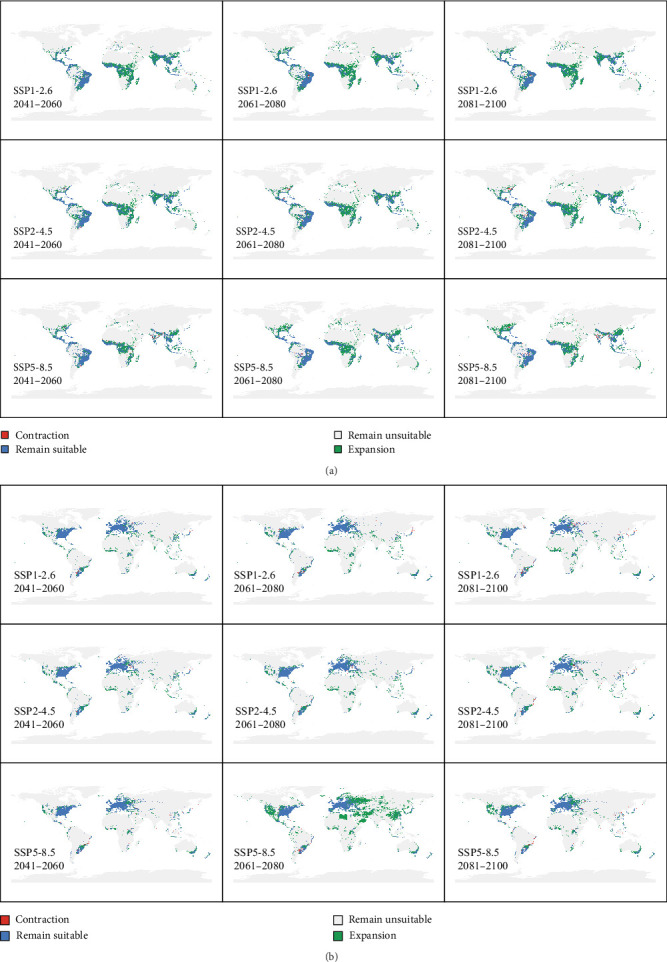
Projected changes in habitat range of (a) *A. aegypti* and (b) *S. calcitrans* under future climate scenarios. Maps show the projected distribution shifts of suitable habitats across three time periods (2050s, 2070s, and 2090s) and three shared socioeconomic pathways (SSP1-2.6, SSP2-4.5, and SSP5-8.5).

To quantify the changes in suitable habitats, we reclassified the continuous outputs into binary maps using the TSS-maximization threshold. The results indicate four types of change: contraction, remain suitable, remain unsuitable, and expansion (Figure 4a,b). The areas of contraction and expansion were also calculated (Table 2).

For *A. aegypti*, the contraction and expansion areas of its suitable habitat range under the SSP1-2.6 and SSP2-4.5 scenarios gradually increase over time, with both the percentage loss and percentage gain showing a steady increase in each scenario. In contrast, under the high-emission SSP5-8.5 scenario, the percentage loss in the 2070s (2.61%) is lower than that in the 2050s (3.77%). A similar trend is observed for *S. calcitrans*, where the percentage loss in the 2070s (2.51%) is lower than that in the 2050s (3.19%) under the SSP5-8.5 scenario.

### 3.4. Variable Importance and Response Curve

**Figure 5:**
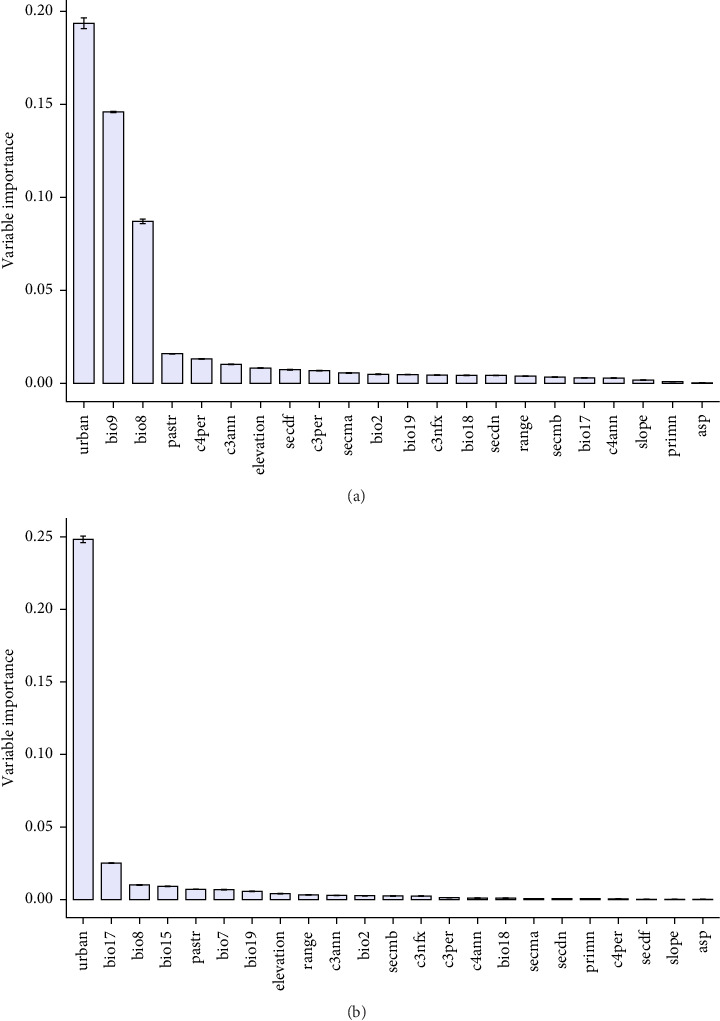
Relative importance of environmental variables contributing to the habitat suitability of (a) *A. aegypti* and (b) *S. calcitrans* under future climate scenarios.

To evaluate the influence of environmental variables on the predicted distribution of *A. aegypti* and *S. calcitrans*, we analyzed variable importance using the output of the model. The results indicate that for *A. aegypti*, urban land was the most important predictor, followed by mean temperature of driest quarter (bio9) and mean temperature of wettest quarter (bio8), managed pasture, and C4 perennial crops also show great importance. For *S. calcitrans*, urban land exhibited the highest importance, followed by precipitation of driest quarter (bio17), mean temperature of wettest quarter (bio8), precipitation seasonality (bio15), and managed pasture (Figure 5a,b).

**Figure 6:**
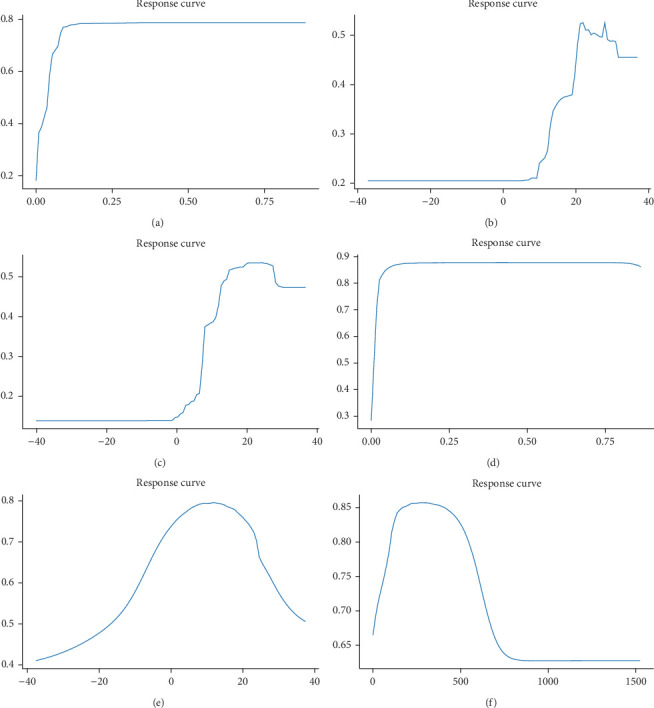
Response curves of the key environmental variables influencing habitat suitability for (a–c) *A. aegypti* and (d–f) *S. calcitrans*. The response curves illustrate how habitat suitability changes along gradients of the most influential variables for *A. aegypti* (urban land, mean temperature of the wettest quarter, mean temperature of the driest quarter) and *S. calcitrans* (urban land, mean temperature of the wettest quarter, precipitation of the driest quarter).

To further explore the relationships between variables and habitat suitability, we generated response curves for the top three most influential variables for each species. The response curve of urban land indicated that, for both *A. aegypti* and *S. calcitrans*, urban land was positively associated with habitat suitability (Figure 6a,d). Additionally, for *A. aegypti*, habitat suitability increased with rising values of bio8 and bio9, indicating a preference for warmer conditions (Figure 6b,c). In contrast, for *S. calcitrans*, habitat suitability exhibited a trend with bio8 and bio17, initially increasing with these variables but decreasing after reaching a threshold (Figure 6e,f).

## 4. Discussion

This study employed SDMs integrated with future climate projections to investigate the potential global distribution and habitat suitability changes for two key vectors of LSD [44, 45, 46, 47, 48]. This significant transboundary animal disease is impacting global livestock industries [49–51]. Utilizing an ensemble forecasting approach, we projected their potential distributions under the 2050s, 2070s, and 2090s, evaluated spatial patterns of habitat contraction and expansion, and analyzed environmental drives through variable importance rankings and response curves. In comparison with the efforts of previous research, this study incorporates the largest sample size of occurrence records for the key LSD vectors. Our findings reveal a pronounced tendency for suitable habitats to shift toward higher latitudes, with varying degrees of change across emission scenarios and time periods, underscoring the potential implications for LSD transmission risk under climate change. These findings warrant further discussion to elucidate their ecological significance and to inform the development of effective disease prevention and vector management strategies.

The key environmental drives identified in this study provide important insights into the potential ecological mechanisms governing LSD transmission risk under climate change. Among the environmental variables, urban land cover exhibited a particularly strong influence on the potential distributions of both *A. aegypti* and *S. calcitrans*, with its contribution even exceeding that of certain climatic factors [52, 53]. As indicated by the response curves, habitat suitability showed a clear positive relationship with the proportion of urban land cover. This finding highlights the critical role of human activities in influencing vector habitats [54, 55]. Notably, *A. aegypti* is known to utilize small artificial water bodies (such as discarded containers, tires, etc.) for oviposition [56, 57], enabling its persistence even in highly urbanized areas with limited natural water sources [58]. Such ecological adaptability to human-modified environments may partly explain the strong association between urban land and *A. aegypti* habitat suitability observed in our models. In addition to urban land cover, the mean temperature of the wettest quarter (bio8) and the mean temperature of the driest quarter (bio9) emerged as important climatic predictors influencing the potential distribution of *A. aegypti*. The response curves indicated that habitat suitability for *A. aegypti* is positively associated with moderate to high values of these variables, suggesting *A. aegypti* requires sufficiently warm conditions during the wet season to support larval development [59, 60], as well as tolerable temperatures during the dry season to ensure adult survival [61]. Such climatic preferences enable *A. aegypti* to exploit a wider environmental niche, particularly under future warming scenarios, where these seasonal temperature thresholds are increasingly likely to be met in higher latitude regions, the distribution of *A. aegypti* may continue to expand polewards [62].

In contrast, the distribution suitability of *S. calcitrans* was not only strongly associated with urban land cover but also with other land-use variables such as managed pasture and crop types, including C3 annual crops and C4 perennial crops. These variables reflect the significance of husbandry systems and agriculture in shaping *S. calcitrans* habitat suitability. The response curves demonstrated that *S. calcitrans* prefers regions with abundant pasture. Aside from providing decaying organic matter and cattle dung as foundational substrates, pastures offer grazing cattle as a blood-feeding source, even breeding facilities near nonfree-range cattle provide favorable conditions for larval development [63, 64]. This habitat preference explains the close relationship between *S. calcitrans* habitat suitability and the intensity of grazing land use, especially in areas with well-developed livestock industries [45, 65]. In addition, climatic variables such as precipitation seasonality (bio15) and precipitation of the driest quarter (bio17) were also influential for *S. calcitrans*. The response curves suggested that the suitable habitats may shift toward areas characterized by lower precipitation seasonality and moderate rainfall during the driest periods, reflecting their dependance on stable moisture conditions for successful reproduction [11, 66]. These findings emphasize that the relevance of key limiting factors may vary across different geographic regions. The distinct habitat preferences of *A. aegypti* and *S. calcitrans* suggest that the transmission dynamics of LSD may differ substantially among regions, depending on the dominant vector species and local patterns of land use [67]. Recognizing these regional differences is crucial for developing regionalized and targeted vector control and disease prevention strategies.

Climate change is widely recognized as one of the primary drivers reshaping species distributions, leading to changes in both the area of habitat expansion or contraction and the geographic location of suitable habitats [68, 69]. Our modeling results reveal potential changes in habitat suitability and distribution patterns of *A. aegypti* and *S. calcitrans* under climate change scenarios. Notably, the divergent trends observed between these two species underscore their distinct ecological responses to climate change. *A. aegypti*, as a typical tropical and subtropical species, possesses strong environmental adaptability and can exploit the diverse habitat resources created by urbanization and human activities. Under global climate change, this species shows a tendency to expand into higher latitudes, reflecting not only a simple range shift but also a climate-driven colonization of previously unoccupied marginal habitats [70, 71], potentially altering the composition of local vector communities and reshaping disease transmission patterns. As temperature and humidity conditions progressively improve in middle and high latitude regions, *A. aegypti* may gain access to new ecological niches, enhancing its potential for successful establishment and growth.

In contrast, *S. calcitrans* exhibits a stronger dependency on specific environmental conditions, particularly pasture systems and stable moisture resources. The expansion of *S. calcitrans* is predicted to be slower than that of *A. aegypti* due to greater moisture constraints [72], leading to a distinct habitat suitability change pattern. In our findings, under the high emission scenario (SSP5-8.5), *S. calcitrans* shows a marked expansion in potential range by the 2070s. However, extreme climatic events, such as enhanced precipitation seasonality and intensified droughts, could reduce the availability of essential breeding substrates [73, 74]. This would weaken its establishment capacity and population persistence in new suitable regions. This ecological limitation is reflected in the sharp reduction of expansion potential projected for the 2090s under SSP5-8.5. Such constraints are commonly observed among livestock-associated disease vectors in tropical and temperate regions, contributing to the increased uncertainty of habitat expansion for this species [75, 76].

Such differences between *A. aegypti* and *S. calcitrans* may influence the spatial pattern of LSD transmission risk. The accelerating process of global urbanization has already significantly weakened vector control capacities and intensified the transmission of vector-borne viruses [77, 78], while climate change further drives the expansion of species distributions and the shift of ecological niches toward higher latitudes [79, 80]. These trends highlight the necessity for future vector-borne disease management strategies to simultaneously incorporate early warning mechanisms for new suitable habitats and systematic management in traditional endemic areas, in order to address the dynamic spatial ecological changes of multiple vector species.

Previous studies on the potential habitat suitability of LSD vectors have primarily focused on single vector species [81, 82], with limited consideration of the potential distribution changes of multiple vectors and their influences on the transmission patterns. This study represents the first attempt to evaluate the habitat suitability dynamics of two vector species with distinct ecological strategies under multiple climate scenarios, providing a novel perspective for understanding the transmission of LSD.

However, this study did not account for possible interspecific interactions, which require more detailed investigations. Such interactions may involve not only competition between *A. aegypti* and *S.calcitrans* for hosts or habitats, but also competition of *A. aegypti* with other *Aedes* subspecies. For instance, *A. aegypti* may compete with *Aedes albopictus*, even with *Culex pipiens* [83, 84]. At present, we are unable to incorporate variables that capture such a relationship. In addition, although we thinned occurrences and generated pseudo-absence points, it is impossible to completely eliminate the influence of sampling bias [85, 86]. Given that *A. aegypti* and *S. calcitrans* serve as vectors for multiple pathogens, including dengue, chikungunya, and others. Understanding their interactions is critical. Therefore, future studies should integrate more comprehensive mechanisms of interspecific competition to further explore the dynamic of vector distributions under climate change. Such efforts will not only address current research gaps but also provide more systematic and forward-looking scientific evidence for global vector-borne disease surveillance and control.

## 5. Conclusion

In conclusion, this study highlights the importance of multiple vector species with distinct ecological strategies when assessing the impacts of climate change on the transmission risk of LSD. Our models revealed distinct habitat expansion patterns of *A. aegypti* and *S. calcitrans* into future environmental changes. These findings suggest that climate change may not only drive habitat shifts of LSD vectors but also change the spatial patterns of LSD transmission risk. Our research provides a novel perspective for improving the prediction of vector-borne disease risks and underscores the necessity of multiple vector assessments, particularly in regions facing emerging risks. Moreover, the development of specific regions, dynamic surveillance and control strategies will be essential to effectively mitigate the risk of LSD transmission in both traditional endemic areas and new emerging regions under climate change.

## Figures and Tables

**Table 1:** Environmental variables selected for modeling.

Candidate variables	Description	Selected For *A. aegypti*	Selected for *S. calcitrans*
Bio1	Annual mean temperature	Yes	Yes
Bio2	Mean diurnal range		
Bio3	Isothermality		

Bio4	Temperature seasonality	—	—

Bio5	Max temperature of the warmest month	—	—

Bio6	Min temperature of the coldest month	—	—

Bio7	Temperature annual range	—	Yes
Bio8	Mean temperature of the wettest quarter	Yes	Yes
Bio9	Mean temperature of the driest quarter	Yes	—
Bio10	Mean temperature of the warmest quarter	—	—
Bio11	Mean temperature of the coldest quarter	—	—
Bio12	Annual precipitation	—	—
Bio13	Precipitation of the wettest month	—	—
Bio14	Precipitation of the driest month	—	—
Bio15	Precipitation seasonality	—	Yes
Bio16	Precipitation of the wettest quarter	—	—
Bio17	Precipitation of the driest quarter	Yes	Yes
Bio18	Precipitation of the warmest quarter	Yes	Yes
Bio19	Precipitation of the coldest quarter	Yes	Yes
C3ann	C3 annual crops	Yes	Yes
C3per	C3 perennial crops	Yes	Yes
C3nfx	C3 nitrogen-fixing crops	Yes	Yes
C4ann	C4 annual crops	Yes	Yes
C4per	C4 perennial crops	Yes	Yes
Primf	Forested primary land	—	—
Primn	Nonforested primary land	Yes	Yes
Secdf	Potentially forested secondary land	Yes	Yes
Secdn	Potentially nonforested secondary land	Yes	Yes
Secma	Secondary mean age	Yes	Yes
Secmb	Secondary mean biomass density	Yes	Yes
Pastr	Managed pasture	Yes	Yes
Range	Rangeland	Yes	Yes
Urban	Urban land	Yes	Yes
Asp	Aspect	Yes	Yes
Slope	Slope	Yes	Yes
Elevation	Elevation	Yes	Yes

**Table 2:** Area changes of suitable habitats for *A. aegypti* (a) and *S. calcitrans* (b) under future climate scenarios.

Scenario	Period	Contraction (km^2^) (percentage change)	Extension (km^2^) (percentage change)
(a)			

	2041–2060	1.56 × 10^5^ (−1.03%)	1.57 × 10^7^ (+96.2%)
SSP1-2.6	2061–2080	1.91 × 10^5^ (−1.20%)	1.68 × 10^7^ (+104.19%)
	2081–2100	2.96 × 10^5^ (−1.86%)	1.70 × 10^7^ (+104.96%)
	2041–2060	2.00 × 10^5^ (−1.32%)	1.53 × 10^7^ (+93.69%)
SSP2-4.5	2061–2080	3.80 × 10^5^ (−2.51%)	1.58 × 10^7^ (+96.70%)
	2081–2100	4.35 × 10^5^ (−2.80%)	1.64 × 10^7^ (+101.25%)
	2041–2060	6.00 × 10^5^ (−3.77%)	1.14 × 10^7^ (+70.45%)
SSP5-8.5	2061–2080	4.16 × 10^5^ (−2.61%)	1.45 × 10^7^ (+90.96%)
	2081–2100	8.22 × 10^5^ (−5.12%)	1.56 × 10^7^ (+93.29%)

(b)			

	2041–2060	2.97 × 10^5^ (−2.10%)	7.01 × 10^6^ (+43.98%)
SSP1-2.6	2061–2080	4.82 × 10^5^ (−3.40%)	7.10 × 10^6^ (+44.62%)
	2081–2100	8.18 × 10^5^ (−5.82%)	6.50 × 10^6^ (+40.76%)
	2041–2060	2.00 × 10^5^ (−1.49%)	7.05 × 10^6^ (+45.15%)
SSP2-4.5	2061–2080	2.50 × 10^5^ (−1.76%)	7.59 × 10^6^ (+48.97%)
	2081–2100	3.75 × 10^5^ (−2.55%)	7.61 × 10^6^ (+49.45%)
	2041–2060	4.95 × 10^5^ (−3.19%)	5.00 × 10^6^ (+33.48%)
SSP5-8.5	2061–2080	4.09 × 10^5^ (−2.51%)	2.14 × 10^7^ (+147.82%)
	2081–2100	5.94 × 10^5^ (−3.10%)	7.68 × 10^6^ (+52.74%)

## Data Availability

The data that support the findings of this study are available upon request from the corresponding author. The data are not publicly available due to privacy or ethical restrictions.
